# A pH-responsive mesoporous silica nanoparticles-based drug delivery system with controlled release of andrographolide for OA treatment

**DOI:** 10.1093/rb/rbab020

**Published:** 2021-06-30

**Authors:** Mingwei He, Zainen Qin, Xiaonan Liang, Xixi He, Bikang Zhu, Zhenhui Lu, Qingjun Wei, Li Zheng

**Affiliations:** 1 Guangxi Engineering Center in Biomedical Material for Tissue and Organ Regeneration, The First Affiliated Hospital of Guangxi Medical University, No. 22 Shuangyong Road, Qingxiu District, Nanning 530021, China; 2 Guangxi Collaborative Innovation Center for Biomedicine, The First Affiliated Hospital of Guangxi Medical University, No. 22 Shuangyong Road, Qingxiu District, Nanning 530021, China; 3 Department of Orthopaedics Trauma and Hand Surgery, The First Affiliated Hospital of Guangxi Medical University, No. 6 Shuangyong Road, Qingxiu District, Nanning 530021, China

**Keywords:** Osteoarthritis, mesoporous silica nanoparticles, polyacrylic acid, pH-response, andrographolide

## Abstract

Andrographolide (AG) has favorable anti-inflammatory and antioxidative capacity. However, it has low bioavailability due to high lipophilicity and can be easily cleared by the synovial fluid after intra-articular injection, leading to low therapeutic efficiency in osteoarthritis (OA). Herein, we designed a nano-sized pH-responsive drug delivery system (DDS) for OA treatment by using modified mesoporous silica nanoparticles (MSNs) with pH-responsive polyacrylic acid (PAA) for loading of AG to form AG@MSNs-PAA nanoplatform. The nanoparticles have uniform size (∼120 nm), high drug loading efficiency (22.38 ± 0.71%) and pH-responsive properties, beneficial to sustained release in OA environment. Compared with AG, AG@MSNs-PAA showed enhanced antiarthritic efficacy and chondro-protective capacity based on IL-1β-stimulated chondrocytes and anterior cruciate ligament transection-induced rat OA model, as demonstrated by lower expression of inflammatory factors and better prevention of proteoglycan loss. Therefore, the AG@MSNs-PAA nanoplatform may be developed as a promising OA-specific and on-demand DDS.

## Introduction

Osteoarthritis (OA), the most general form of arthritis, has been recognized as a multifaceted joint disorder affecting billions of people worldwide [1, 2]. Commonly prescribed medications for OA include non-steroidal anti-inflammatory drugs (NSAID), corticosteroids, and analgesics, which only contributed to symptomatic relief but can hardly restrain the development of OA [3]. In addition, they have serious side effects, like gastrointestinal bleeding, cardiovascular disease, etc [4]. Therefore, it is imperative to find an alternative for OA therapy.

Plant-derived natural products attract most attention in the treatment of inflammatory disease for their widespread use in traditional and folk medicine. *Andrographis panicula* has long been utilized as a folk remedy for the therapy of inflammation, fever, and other infectious diseases in China, Korea, Japan, and India [5]. As the major plant extract of *A. panicula*, Andrographolide (AG) is currently prescribed for the treatment of inflammation-related diseases, upper respiratory tract infection, laryngitis, and antihepatitis in China [6–9]. It was known to possess the antioxidant and anti-inflammatory efficiency on cartilage [10, 11]. A recent clinical study reported the efficiency of AG in the relieving of rheumatoid arthritis symptoms with reduced immunological markers after 14-week treatment [12]. However, the low aqueous solubility and high lipophilicity of AG limited its bioavailability and thus decreased the pharmacological effects after oral administration [13].

Recently, nano-size drug delivery system (DDS) for intra-articular (IA) injection attracts most attention, which can increase the retention of drugs in joint by improving the hydroscopicity and thus the bioavailability [14–16]. Combination of biocompatible inorganic nanoparticles with hydrophobic drugs is one of the strategies in DDS [17]. Among various inorganic nanoparticles, mesoporous silica nanoparticles (MSNs) with highly ordered mesoporous structure that can provide extremely high specific surface area and aperture volume are suitable for drug loading. And the well-defined surface of MSNs allows the conjunction with pore-blocking materials to prevent premature cargo release of drug, or with stimuli-responsive ligands for controlled release, or with specific ligands for targeting. MSNs as drug delivery vehicle have been widely employed in biomedical fields [18–20]. For OA treatment, the phospholipid-coated MSNs as nanocarriers exhibited excellent lubrication capability [21]. Nevertheless, current MSNs-based carriers are limited for clinical application because most of them have low drug release rate and disatisfiable responsiveness to OA environment that may decrease the therapeutic efficiency.

The metabolic activity of cells in the inflammatory site is increased which resulted in hypoxia and induces a shift to anaerobic glycolysis and lactification [22]. The formation of lactate acidifies the microenvironment, such as the pH as low as 6.0 have been defined in the articular cavity of patients with arthritis [23]. A pH-responsive DDS which could minimize unnecessary drug release under the low inflammatory activity, thereby prolonging the joint residence time and the duration of the therapeutic effectiveness may be helpful for OA treatment.

Herein, we developed a pH-responsive nano-size DDS (MSN) loaded with AG for OA therapy. In this system, MSNs were firstly modified with polyacrylic acid (PAA) to form MSNs-PAA, in which PAA served as pore-blocking agent and pH-sensitive ligand since PAA can disassemble in acid environment while is stable at physiological pH (∼7.4) [24]. Then, AG@MSNs-PAA was prepared by loading AG with MSNs-PAA. It is expected that in response to the acid OA environment, AG@MSNs-PAA disassemble to expose the pore of MSNs for on-demand release of AG. The anti-inflammatory efficiency of the pH-responsive nanocarriers was investigated in IL-1β-induced chondrocytes and anterior cruciate ligament transection (ACLT)-induced rat OA model. This study may provide a reference for the design and synthesis of novel nano-DDS in OA therapy.

## Materials and methods

### Fabrication of MSNs

MSNs were synthesized according to the study reported previously [25]. About 0.2 g of cetyltrimethylammonium bromide (CTAB) was added into 96 ml of ultrapure water using the ultrasonic method for dissolving followed by adjusting pH = 11.0 by adding 1.1 ml of ammonia water (29%). The solution was stirred at 80 °C for 30 min. Subsequently, the above mixture was mixed with 1 ml of tetraethyl orthosilicate by adding dropwise and stirred at 80°C for 2 h. The resultant white precipitate was obtained by vacuum filtration and successively rinsed with deionized water and alcohol for three times. Then, the removal of the template (CTAB) was performed by dispersing precipitate in 20 ml of methanol and 0.2 ml of concentrated hydrochloric acid and then refluxed at 60 °C for 6 h. The MSNs were collected by vacuum filtration and dried in a vacuum drying chamber (Boxun, Shanghai, China) oven overnight.

### Preparation of PAA functionalized MSN (MSNs-PAA)

To obtain the MSNs-PAA, amino-functionalized MSNs was prepared by dispersed 0.2 g of MSNs in 20 ml methyl alcohol followed by adding 40 µl of 3-aminopropyltriethoxysilane and stirred at room temperature for 24 h. Then 20 mg of amino-functionalized MSNs was diffused in 2 ml of ultra-pure water using sonication and mixed with 4 ml activatory aqueous solution containing 1-Ethyl-3-(3-dimethylaminopropyl)carbodiimide hydrochloride (EDC) (0.04 g), N-Hydroxysuccinimide (NHS) (0.08 g) and PAA (30 mg) using a stir at room temperature overnight. MSNs-PAA was harvested by centrifuged (12 000 rpm, 10 min), then the unreacted reagents were removed by washing with deionized water and the sample dried by a vacuum drying chamber.

### Ag loading

AG was purchased from Chengdu Must Bio-technology Co. LTD. (Sichuan, China). For AG loading, 10 ml of methanol was used for dispersing 100 mg of MSNs by using a sonicator. Then, added with 5 ml of AG (3 mg/mL, dissolved in methanol) solution and stirred at 25 °C overnight. The methanol was removed rotary evaporator at 50 °C. The nanocarriers that loaded with AG were dispersed in aqua destillata and centrifuged (12 000 rpm, 10 min) for the removing of any unloaded AG. The collected AG@MSNs was then dried in a vacuum oven overnight. The grafting of PAA was subsequently performed by the procedure described above to obtain AG@MSNs-PAA.

The amount of AG remaining in the washing solution was quantified by high-performance liquid chromatography (HPLC; BioTek, USA) with a LiChrospher and capped column (125 mm × 4 mm, particle size 5 µm). The drug encapsulation efficiency (EE, %) and drug loading capacity (LC, %) were calculated according to the formulas as follow:

(1) $$\mathrm{EE}\;\left(\%\right)=\frac{\text{initial}\;\text{amount}\;\text{of}\;\text{AG}–\text{residual}\;\text{AG}}{\text{initial}\;\text{amount}\;\text{of}\;\text{AG}}\times 100\%$$

(2) $$\mathrm{LC}\;\left(\;\%\right)=\frac{\text{initial}\;\text{amount}\;\text{of}\;\text{AG}–\text{residual}\;\text{AG}}{\;\text{amount}\;\text{of}\;\text{MSNs}}\times 100\%.$$

### Characterization of nanoparticles

The morphology and size of MSNs and MSNs-PAA were visualized via a transmission electron microscope (TEM; FEI, USA) at an operating voltage of 200 kV. Dynamic light scattering (DLS) was performed to analyze the particle size of MSNs and MSNs-PAA was further analyzed by using a Laser Particle Size Analyzer (Malvin, UK). The Fourier Transform Infrared Spectrum (FT-IR) of MSNs, and MSNs-PAA was recorded by an infrared spectrometer (PerkinElmer, USA) within the wavelength range from 400 to 4000 cm^−1^. The phase structure of MSNs and MSNs-PAA was characterized using an X-ray Diffraction (XRD; Rigaku, Japan).

### In vitro AG release

To explore the AG release from nanoparticles, 8 mg of AG@MSNs or AG@MSNs-PAA was placed into dialysis bags and immersed in 20 ml pH = 5.6 or pH =7.4 phosphate buffer saline (PBS), then shake at 100 rpm and kept in 37 °C for 72 h. The concentration of released AG in the buffer was measured using the HPLC at selected time points. Isopyknic fresh PBS was replenished into the dialysis system.

### Cell culture

Chondrocytes of Sprague Dawley (SD) rats were separated, as reported previously [26]. Briefly, articular cartilage was collected from the terminal of tibia and femur. Any other tissue around the cartilage was removed by digesting with 0.25% of trypsin-ethylenediaminetetraacetate (EDTA) (Solarbio, Beijing, China) for 30 min. Subsequently, 2 mg/ml collagenase II (Sigma, USA) was used to digest the cartilage. After 4 h of digestion, cells were harvested and cultured in Dulbecco's Modified Eagle’s Medium-High glucose (DMEM, Hyclone, USA) that supplemented with 1% penicillin/streptomycin (Solarbio, Beijing, China) and 10% fetal bovine serum (Hyclone, USA).

### Cytotoxicity test

The toxicity of MSNs-PAA and AG@MSNs-PAA on chondrocytes was assessed by a Cell Counting Kit-8 (CCK-8, Dojindo Kagaku, Japan). Chondrocytes were put into a 96-well plate at a density of 5000 cells/well. After that, the chondrocytes were treated with various concentrations of MSNs-PAA or AG@MSNs-PAA for 3 days. The culture medium was refreshed with serum-free medium and added with 10 μL CCK-8 for 4 h. A microplate reader (Thermo Fisher Scientific, USA) was utilized for the measurement of the absorbance at 450 nm.

### Nanoparticle treatment

To better understand the pH-sensitive potential of nanocarriers, a partial acidic culture condition (pH = 6.8) was utilized to simulate the OA environment to study the anti-inflammatory effect of AG@MSNs-PAA on IL-1β-induced chondrocytes *in vitro*. Chondrocytes were divided into four groups: (1) Control group: with culture medium only; (2) IL-1β group: with IL-1β (PeproTech, USA, 10 ng/mL) for 24 h; (3) AG group: with 10 ng/mL IL-1β for 24 h after pre-incubating with 8 µM AG for 1 h; (4) AG@MSNs-PAA group: with 10 ng/mL IL-1β for 24 h after pre-incubating with AG@MSNs-PAA (containing 8 µM of AG) for 1 h.

### Live/dead cells assay

Live/dead cells assessment was performed by utilizing a live/dead viability assay kit (Thermo Fisher Scientific, USA). Chondrocytes were rinsed with PBS and incubated with the solution containing 2 μM of calcein acetoxymethyl ester and 4 μM of ethidium homodimer-1 for 30 min at room temperature in the dark. As described in the manufacturer’s protocol, the viable cells were dyed green and the dead cells were dyed red. After washed with PBS again, the cells were photographed by a fluorescent microscope (OLYMPUS, Japan).

### Biochemistry assay

Chondrocytes were digested with 50 μg/mL proteinase K (Boster, Wuhan, China) at 56°C for 16 h. The lysate was incubated with Hoechst 33258 (Thermo Fisher Scientific, USA) for detecting the DNA content which was normalized by the stander curve of calf thymus DNA. For GAG content, 1,9-dimethylmethylene blue (DMMB, Sigma, USA) method was used. DMMB and cell lysate were mixed and incubated for 5 min. Then the absorbance at 525 nm was measured by a plate reader and calculated according to the standard curve of chondroitin sulfate (Sigma, USA). GAG content was then normalized to the total DNA content.

### Quantitative real-time polymerase chain reaction (qRT-PCR)

The qRT-PCR was performed according to the study reported previously [27]. The primer sequences used for qRT-PCR were presented in Table 1.

**Table 1.** Primer sequences used in qRT-PCR experiments

Gene	Forward primer	Reverse primer
*Col2α1*	5′-GTCCTACAATGTCAGGGCCA-3′	5′-ACCCCTCTCTCCCTTGTCAC-3′
*Acan*	5′-GAATGGGAGCCAGCCTACAC-3′	5′-GAGAGGCAGAGGGACTTTCG-3′
*Mmp3*	5′-GGCTGTGTGCTCATCCTACC-3′	5′-TGGAAAGGTACTGAAGCCACC-3′
*Mmp13*	5′-GGACAAAGACTATCCCCGCC-3′	5′-GGCATGACTCTCACAATGCG-3′
*Gapdh*	5′-TCCAGTATGACTCTACCCACG-3′	5′-CACGACATACTCAGCACCAG-3′

### 2.12 Animal procedure

A total of 56 male SD rats obtained from Animal Research Committee of Guangxi Medical University (Nanning, China) (10-week-old, weight 200 ± 10 g) were used in the animal experiments. All procedures were carried out according to the guide for the care and use of laboratory animals approved by the Experimental Animal Ethics Committee of Guangxi Medical University.

SD rat OA model was surgically induced by ACLT according to previous reports [28]. Forty-eight rats were anesthetized by intraperitoneal injection of pentobarbital sodium (30 mg/kg body weight) and underwent bilateral ACLT on the knee joints to induce OA. The other six were received sham operations (normal group), of which the articular cavity was opened and sutured with the short anterior cruciate ligament intact. After surgery for 4 weeks, the rats were randomly divided into three groups: NS group (intra-articular (IA) injection with 0.5 ml of saline); AG group (IA injections of 0.5 ml of 8 µM AG); AG@MSNs group (IA injections with 0.5 ml of 8 µM AG@MSNs); AG@MSNs-PAA group (IA injections with 0.5 ml of 8 µM AG@MSNs-PAA). IA injections were performed once a week. The animals in these groups were sacrificed for further analysis at 4 and 8 weeks after therapy.

### Macroscopic assessment

SD rats were intraperitoneal injected with an overdose of pentobarbital to sacrifice after 4 and 8 weeks of therapy. The knee joints were collected and the alteration in cartilage were observed and evaluated by three independent observers who were blinded to the treatment groups based on a scale put forwarded by Pelletier *et al.* [29].

### Histological evaluation

For the *in vitro* study, cells in each group were rinsed with PBS and fixed with 95% alcohol for 30 min. Then, 0.1% safranin O (Sigma, USA) was used to stain the cells for 10 min and any residual dye was removed by washing with water. Images were obtained by a microscope (Olympus, Japan).

For the *in vivo* study, the total knee joints were fixed with 4% paraformaldehyde for at least 2 days after macroscopic analysis, then 10% EDTA and an ultrasonic instrument was used for decalcification for 2 weeks. The samples were embedded in paraffin and subsequently cut into 3 μm sections. Paraffin sections were de-waxed and stained with hematoxylin-eosin (HE, JianCheng Biotech, China) or safranin O/fast green (Solarbio, Beijing, China) for histological examination. Each section was scored separately by three independent researchers based on the scale reported by Osteoarthritis Research Society International (OARSI) [30].

### Immunofluorescence staining

Immunofluorescence staining was performed to observe the expression of MMP1 *in vitro*. The cells and dewaxed sections were rinsed with PBS and exposed to hydrogen peroxide (H_2_O_2_; Sangon Biotech, Shanghai, China; 3% (v/v)) for 15 min at room temperature to block any endogenous peroxidase activity. After blocking with normal goat serum for 20 min at room temperature, samples were incubated with primary antibody against MMP1 (1:200) at 4°C overnight. Samples were treated with fluorescence second antibody for 1 h and then 4',6-diamidino-2-phenylindole (DAPI, Solarbio, Beijing China) was used for staining the nucleuses. The photographs were captured using a fluorescence microscope (Leica, Germany).

### Statistical analysis

IBM SPSS statistics 20.0 was carried out to perform the statistical analyses. All the experiments were repeated at least three times, and data were presented as mean ± SD. The multiple comparison tests were analyzed by one-way analysis of variance (ANOVA). *P *<* *0.05 was considered as statistical significance.

## Results

### Characterization of MSNs and MSNs-PAA

**Figure 1.**
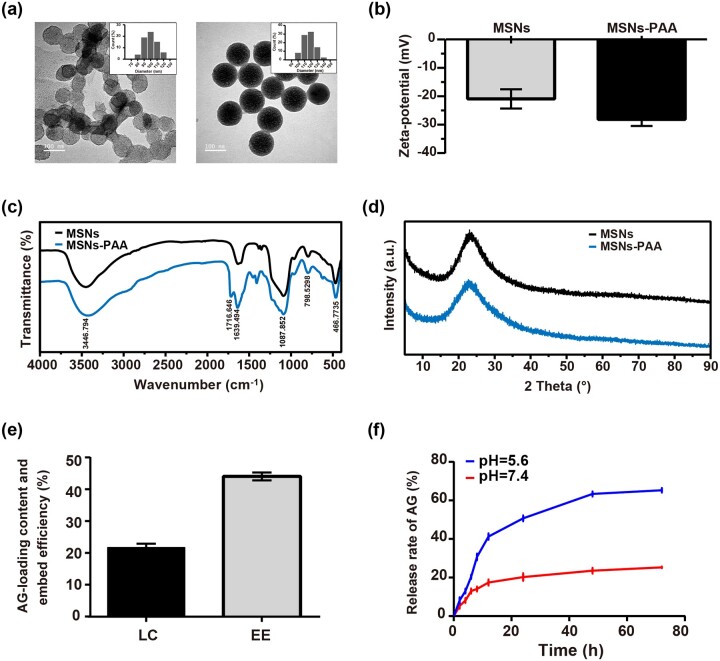
Characterization of nanoparticles. (**a**) TEM images and size distribution (insets) of MSNs and MSNs-PAA (scale bar: 100 nm). (**b**) Zeta potential, (**c**) FT-IR spectra, and (**d**) XRD spectra of MSNs and MSNs-PAA. (**e**) Drug loading capacity and encapsulation efficiency of MSNs-PAA. (**f**) Cumulative drug release curve of AG@MSNs-PAA at pH 5.6 or 7.4 *in vitro*. Data were shown as mean ± SD (*n* = 3).

TEM (Fig. 1a) and DLS (Fig. 1a insets) were performed to analyze the morphology and size of MSNs and MSNs-PAA. The ordered mesoporous and rough surface on MSNs was shown. When grated with PAA, the surface became smooth and the ordered mesoporous structure disappeared. The size of MSNs is about 100 nm and became bigger when coated with PAA of which the size is about 120 nm. The Zeta potential was changed from −20.93 ± 3.40 mV for MSNs to −28.12 ± 2.35 mV for MSNs-PAA, suggesting the successful grating PAA to MSNs (Fig. 1b). Successful conjugation of PAA to MSNs was also confirmed by FT-IR (Fig. 1c). The vibration bands in the 490–1090 cm^−1^ range indicates that the typical of pure silica materials were exhibited in both the spectra of MSNs and MSNs-PAA. The peaks at 1639.49 cm^−1^ and 1716.64 cm^−1^ are contributed to the C = O stretching vibration, suggesting the successful PAA grafting on the surface of MSNs. The grafting of PAA did not change the crystal form of MSNs, as evidenced by the same location of the diffraction peak in MSNs and MSNs-PAA (Fig. 1d).

### Ag encapsulation capacity and in vitro release study

Prior to the initiation of the *in vitro* drug release studies, the drug EE and drug LC of AG@MSNs-PAA were measured. As calculated by HPLC, the LC and EE of AG@MSNs-PAA are 22.38 ± 0.71% and 43.39 ± 0.33% (Fig. 1e).

AG release profiles from AG@MSNs-PAA *in vitro* were evaluated in buffer solution that simulated the physiologic pH (7.4) and the osteoarthritic pH (5.6). AG release kinetics of AG@MSNs-PAA at different pH was shown in Fig. 1f. At pH = 7.4, an initial burst release was shown at the first 12 h and only about 24% of AG was released. While at pH = 5.6, the gradually AG release was sustained for 48 h and the cumulative release rate is about 80%.

### Ag@MSN-PAA promote the viability of chondrocytes

**Figure 2.**
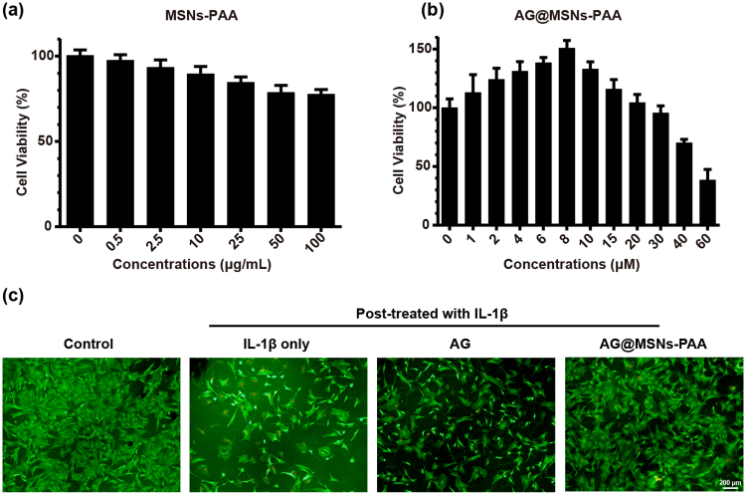
Effect of nanoparticles on cell viability. (**a**, **b**) Cytotoxicity of MSNs-PAA and AG@MSNs-PAA on chondrocytes. (**c**) Cell viability of IL-β1-induced chondrocytes that treated with AG, AG@MSNs, or AG@MSNs-PAA for 24 h (scale bar: 200 μm). Data were represented as mean ± SD (*n* = 3).

CCK8 assessment was performed to detect the biocompatibility of MSNs-PAA and AG@MSNs-PAA. As shown in Fig. 2a, MSNs-PAA has demonstrated almost no toxicity at concentrations range from 0 to100 µg/ml. AG@MSNs-PAA promoted cell proliferation at concentrations below 20 µM, at a concentration of 8 µM exhibited the highest cell viability (Fig. 2b). Therefore, 8 µM of AG and AG@MSNs-PAA were applied for the subsequent experiments.

The live/dead assay revealed that cell viability was obviously decreased with the stimulation of IL-1β, as evidenced by more dead cells and less viable cells (Fig. 2c). In the treatment of AG or AG@MSNs-PAA, the cell viability was significantly increased. Particularly in the AG@MSNs-PAA group, more viable cells were shown compared to AG.

### Ag@MSNs-PAA protects chondrocytes from IL-1β-stimulated destruction in vitro

**Figure 3.**
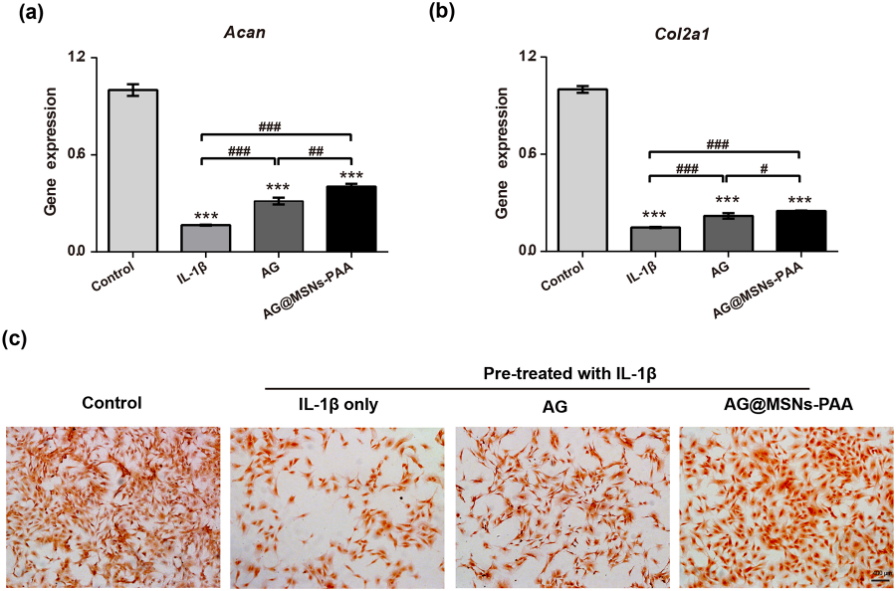
Chondro-protective effects of nanoparticles on IL-1β-induced chondrocytes. (**a**, **b**) qRT-PCR was performed to detect the expression levels of chondrogenic markers *Col2α1* and *Acan*. (**c**) Quantification of GAG content. (**d**) Safranin O stained for GAG production (scale bar, 400 µm). Data were represented as the mean ± SD of three independent culture experiments (*n* = 3). ^#^*P *<* *0.05; ^##^*P *<* *0.01; ***^, ###^*P *<* *0.001.

Gene expression levels of cartilage-specific markers including *Acan* and *Col2α1* were analyzed by qRT-PCR to explore the effect of AG@MSNs-PAA on chondrocytes. The levels of *Col2α1*and *Acan* were notably increased by the stimulation of IL-1β (Fig. 3a and b). AG or AG@MSNs-PAA remarkably decreased the damage in chondrocytes that stimulated by IL-1β, as evidenced by higher expression of cartilage-specific markers. Furthermore, among the two AG-treated groups, most minimized changes in phenotype loss of chondrocytes were shown in AG@MSNs-PAA group.

To further verify the effect of AG@MSNs-PAA on the protection of chondrocytes, safranin O staining which stained the GAG (a main component of ECM of chondrocytes) was detected (Fig. 3c). IL-1β treatment caused obvious GAG loss compared with normal group. AG showed an inhibitory effect on GAG loss compared to the IL-1β group. AG@MSNs-PAA had the most significant potent inhibition on GAG loss.

### Ag@MSNs-PAA prevents IL-1β-induced inflammation in vitro

**Figure 4.**
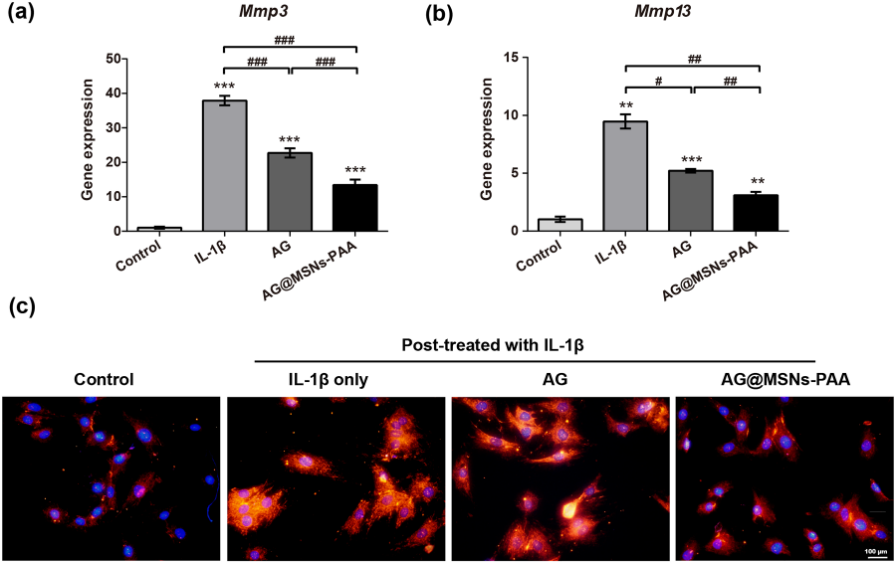
AG@MSNs-PAA prevents IL-1β-induced inflammation in chondrocytes *in vitro*. (**a**, **b**) Relative mRNA levels of OA relative genes (*Mmp3 and Mmp13*). (**c**) MMP13 expression was analyzed by immunofluorescence staining (scale bar: 100 µm). Data were presented as the mean ± SD (*n* = 3). ^#^*P *<* *0.05; **^, ##^*P *<* *0.01; ***^, ###^*P *<* *0.001.

Expression levels of *Mmp3* and *Mmp13* mRNA was detected to assess the potential impact of AG@MSNs-PAA on inflammation that stimulated by IL-1β *in vitro*. In the stimulation of IL-1β, the expression of inflammatory makers including *Mmp3* and *Mmp13* were significantly up-regulated (Fig. 4a and b). AG and AG@MSNs-PAA prevented the IL-1β-mediated upregulation of *Mmp3* and *Mmp13*. Comparatively, although AG inhibited the expression of inflammatory makers, its effect is not so marked as the nanocarriers. AG@MSNs-PAA showed most extensive suppression in the expression of *Mmp3* and *Mmp13* by 64.66 and 67.33%, respectively.

The expression of OA-specific marker MMP13 was detected by immunofluorescent staining. IL-1β obviously increased the expression of MMP13 in chondrocytes, and it was attenuated when the cells were pretreated with AG or AG@MSNs-PAA (Fig. 4c), this was in accordance with that in inflammatory markers expression (Fig. 4b). Results indicated that controlled released of AG may potent suppress the inflammatory reaction in chondrocytes that activated by IL-1β.

### Ag@MSNs-PAA alleviates inflammation and cartilage destruction in vivo

**Figure 5.**
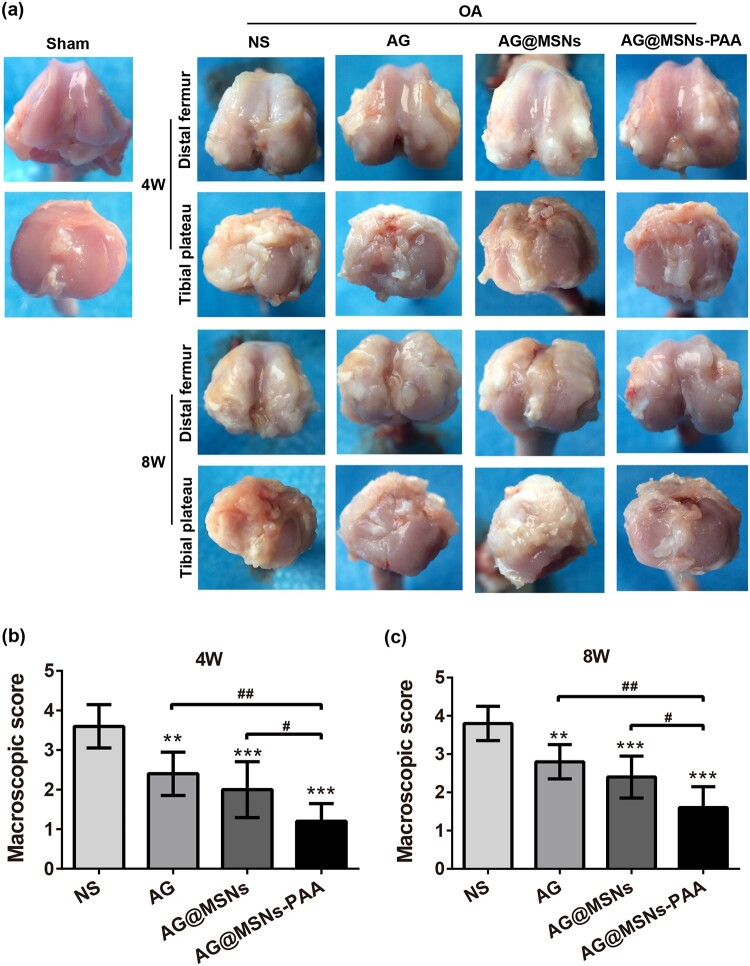
AG@MSNs-PAA attenuates osteophyte formation and cartilage erosion *in vivo*. (**a**) Macroscopic appearance of distal femur and tibial Plateau from rats after treatment for 4 and 8 weeks. (**b**, **c**) Macroscopic scores for macroscopic changes of articular cartilage from rats after treatment for 4 and 8 weeks. Data were represented as the mean ± SD (*n* = 5). ^#^*P *<* *0.05; **^, ##^*P *<* *0.01; ***P <* *0.001.

The changes of articular cartilage in each group at 4 and 8 weeks post-injection was evaluated by macroscopic evaluation. In the sham group, no obvious macroscopic cartilage abrasion was observed (Fig. 5a). OA features including osteophyte formation as well as cartilage surface erosion was exhibited in all OA-induced groups. Especially in the NS group, massive osteophyte formation and a large area of cartilage erosion were observed though AI injection of the AG or AG@MSNs could somewhat ameliorate the destruction in cartilage; the characteristics of OA were still manifestative. AG@MSNs-PAA displayed minimal changes in cartilage compared to the other three ACLT groups. Macroscopic score further verifies the protective effect of AG@MSNs-PAA on cartilage, of which the score was decreased by 66.67 and 57.89% at 4 and 8 weeks compared to NS (Fig. 5b and c). However, the score in AG and AG@MSNs was decreased of 33.33 and 44.44% at week 4, 26.32 and 36.84% at week 8 in comparison to NS.

**Figure 6.**
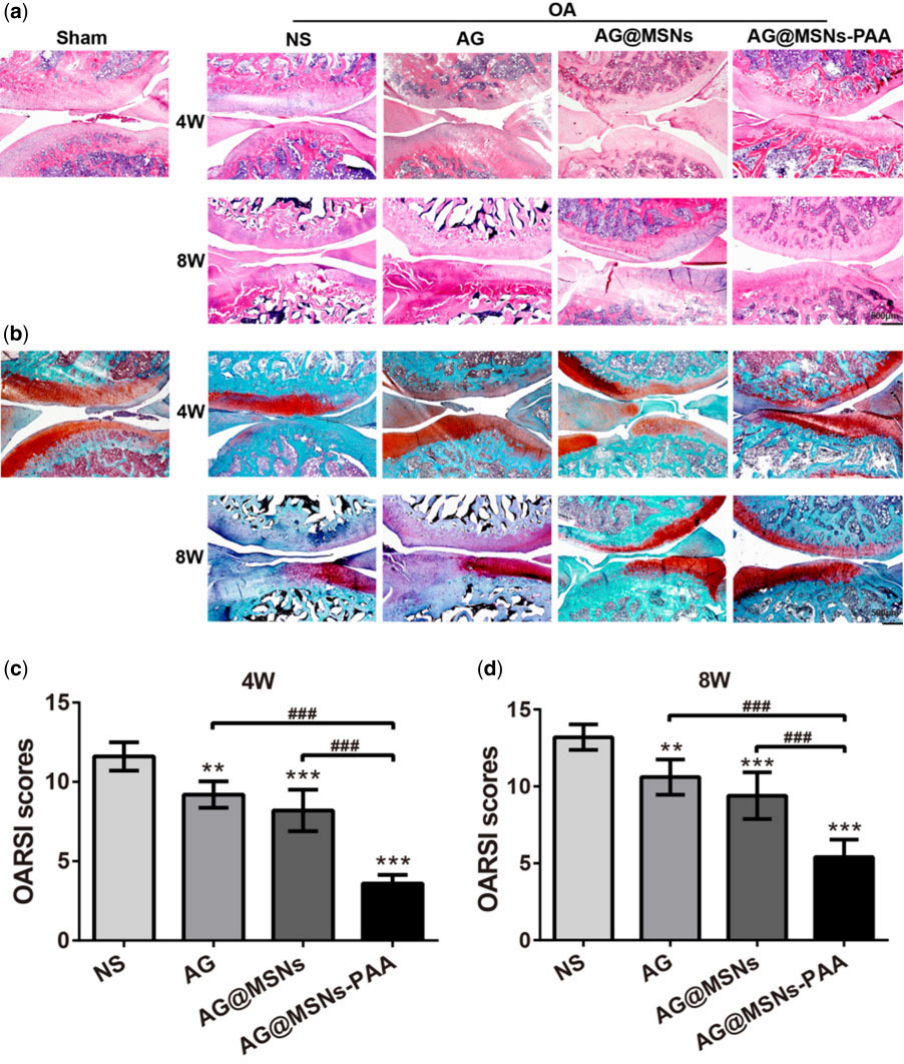
AG@MSNs-PAA prevents cartilage changes induced by ACLT in OA rats. (**a**, **b**) Cartilage was stained by hematoxylin and eosin (HE) and safranin O/fast green after treatment for 4 and 8 weeks (scale bar: 500 nm). (**c**, **d**) OARSI scores for histological changes of articular cartilage after treatment for 4 and 8 weeks. Data were represented as the mean ± SD (*n* = 5). **P <* *0.01; ***^,###^*P *<* *0.001.

The results of histologic assessment were consistent with that in the macroscopic evaluation (Fig. 6). In NS group, progressive cartilage degeneration and superficial fibrillation were observed and gone worse over time. In AG and AG@MSNs groups, matrix vertical fissures, thinner cartilage as well as minor surface destabilization were observed. However, in the treatment of AG@MSNs-PAA, even at week 8 only mild superficial fibrillation and generally intact surfaces were exhibited (Fig. 6a and b). In addition, Ai-injection of AG, AG@MSNs and AG@MSNs-PAA significantly decreased OARSI scores to 20.69, 29.31 and 68.97% at week 4 and 19.70, 28.79, and 59.10% at week 8 compared to NS group (Fig. 6c and d).

## Discussion

The application of AG for nonsurgical IA treatments of OA is limited by its hydrophobicity and rapid clearance rate. Nano-size DDS has been developed as promising therapeutic strategies for IA injection to improve the absorption and joint retention. However, it still remains inadequate for the low drug loading capacity and release rate which resulted in poor therapeutic efficiency. Herein, we developed a pH-responsive nanocarrier (AG@MSNs-PAA) by grafting a pH-sensitive PAA on MSNs with loaded AG for bioresponse and sustained release of therapeutic agents for the treatment of OA.

MSNs with excellent biocompatibility, large pore volume, and versatile possibility for modification, are promising DDS for drug delivery. Its high surface area and interior volume allow for easy loading with high content of hydrophobic cargos from a nonaqueous solution and be retained in aqueous environments [31]. It was reported that the drug LC of MSNs were higher than that in the other carriers such as polymersomes and liposomes [32]. In our study, the LC of AG@MSNs-PAA was 22.38 ± 0.71% (Fig. 1e), which was higher than that encapsulated in micelles (11.2 ± 0.40%) [33]. Drug delivered into the cells by MSNs mainly through endocytosis or macropinocytosis. When internalized, it is preconceived that lipid membrane components enhance the phase transfer of the encapsulated hydrophobic payload allowing to be gradually released [31].

As a natural extracted molecular, AG has been reported to prevent the degeneration of human nucleus pulposus cells and protect chondrocytes from destruction that mediated by oxidative stress through activating the Keap1–Nrf2–Are signaling pathway [10, 34]. AG and its derivatives also decrease serum iNOS activity, NO production, and PGE production in arthritis [35]. However, the hydrophobic property of AG may contribute to poor bioavailability. In addition, it can be quickly cleared by joint fluid when AI injection. Encapsulation of AG into MSNs for controlled release may prolong the retention time of AG in the joint to enhance therapeutic efficiency. As expected, MSNs-PAA-based delivery system showed gradual release of AG (Fig. 1f). Controlled release of AG from MSNs-PAA significantly prevented the IL-1β-induced chondrocytes from destruction compared to AG alone, as evidenced by superior cell viability (Fig. 2c), higher expression levels of cartilage specific makers (Fig. 3a and b), and long-term inhibition of GAG loss (Fig. 3c). Simultaneously, sustained release of AG led to inhibit the level of inflammatory reaction (Fig. 4). Furthermore, the cartilage degradation and GAG loss *in vivo* had no significant difference between IA injection of AG@MSNs and AG alone (Figs 5 and 6), suggesting that the encapsulation of AG in MSNs for sustained release and MSNs itself could not improve the therapeutic efficiency to attenuate the progress of OA.

PH-responsive AG@MSNs-PAA modified by PAA showed better therapeutic efficiency in OA. PAA was successfully grafted on the surface of MSNs for pore blocking (Fig. 1a), and the *in vitro* drug release proﬁles of AG@MSNs-PAA showed better release under acidic condition (pH = 5.6) than neutral condition (pH = 7.4) (Fig. 1g). MSNs functionalized with PAA possess the pH-responsive property for on demand release of AG to exert chondro-protective (Fig. 3) and anti-arthritic effect (Figs 4–6) both *in vitro* and *in vivo*. As indicated by PCR, mRNA level of *Cola2A1* and *Acan* in chondrocytes were 13.85 and 28.53% higher in the treatment of AG@MSNs-PAA than that in AG (Fig. 3a and b). The GAG loss in IL-1β-stimulated chondrocytes and ACLT-induced OA model was also distinctly rescued by the treatment of AG@MSNs-PAA compared to AG and AG@MSNs (Figs 3c and d, 6b). In the comparison to AG, AG@MSNs-PAA also decreased the expression of pro-inflammatory factors, including *Mmp3* and *Mmp13* by 41.02% and 40.64% (Fig. 4a and b). Additionally, the cartilage degradation was suppressed by IA-injection of PAA-functionalized MSNs, with 33.33 and 42.55% decrease in macroscopic score and OARSI score at week 8 when compared with AG@MSNs (Figs 5c and 6d).

## Conclusions

In summary, we fabricated a pH-responsive DDS by using MSNs nanoparticles grafted with PAA and loaded with AG for OA treatment based on the IL-1β-stimulated chondrocytes and an ACLT-induced OA model. AG@MSNs-PAA could respond to the acidic environment of OA in joints for sustained release to match the disease activity and effectively attenuate the development of OA. This study provides a reference for design of on-demand drug releasing systems for OA therapy.

## Funding

This study was financially supported by The National key research and development program of China (2016YFB0700800), the National Natural Science Foundation of China (Grant No. 81972120), the Guangxi Science and Technology Base and Talent Special Project (Grant No. GuikeAD19254003), the Seventh Batch of Special Experts in Guangxi (Professor Wei Yao).

*Conflict of interest statement*. The authors declare no conflict of interests.
